# Total, Bioavailable, and Free 25-Hydroxyvitamin D Equally Associate with Adiposity Markers and Metabolic Traits in Mexican Adults

**DOI:** 10.3390/nu13103320

**Published:** 2021-09-23

**Authors:** Berenice Rivera-Paredez, Alberto Hidalgo-Bravo, Guadalupe León-Reyes, Leith S. León-Maldonado, Arnoldo Aquino-Gálvez, Manuel Castillejos-López, Edgar Denova-Gutiérrez, Yvonne N. Flores, Jorge Salmerón, Rafael Velázquez-Cruz

**Affiliations:** 1 Research Center in Policies, Population and Health, School of Medicine, National Autonomous University of Mexico (UNAM), Mexico City 04510, Mexico; bereriveraparedez7@gmail.com (B.R.-P.); jorge.salmec@gmail.com (J.S.); 2 Department of Genetics, National Institute of Rehabilitation (INR), Mexico City 014389, Mexico; dr_genetica@yahoo.com; 3 Genomics of Bone Metabolism Laboratory, National Institute of Genomic Medicine (INMEGEN), Mexico City 14610, Mexico; greyes@inmegen.gob.mx; 4 National Council for Science and Technology (CONACyT)—Center for Population Health Research, National Institute of Public Health (INSP), Cuernavaca 62100, Morelos, Mexico; lmleiths@gmail.com; 5 National Institute of Respiratory Diseases “Ismael Cosío Villegas” (INER), Mexico City 14080, Mexico; araquiga@yahoo.com.mx (A.A.-G.); mcastillejos@gmail.com (M.C.-L.); 6 Nutrition and Health Research Center, National Institute of Public Health (INSP), Cuernavaca 62000, Morelos, Mexico; edgar.denova@insp.mx; 7 Epidemiological and Health Services Research Unit, Mexican Institute of Social Security, Cuernavaca 62000, Morelos, Mexico; ynflores@ucla.edu; 8 UCLA Department of Health Policy and Management and Kaiser Permanente Center for Health Equity, Fielding School of Public Health, Los Angeles, CA 90095, USA; 9 UCLA Cancer Prevention and Control Research Center, Fielding School of Public Health and Jonsson Comprehensive Cancer Center, Los Angeles, CA 90095, USA

**Keywords:** total 25-hydroxyvitamin D, bioavailable 25-hydroxyvitamin D, free 25-hydroxyvitamin D, adiposity, metabolic traits

## Abstract

Epidemiological studies suggest a relationship between total 25-hydroxyvitamin D [25(OH)D], adiposity, and metabolic traits. The bioavailability of 25(OH)D is regulated by the albumin, vitamin D binding protein (VDBP), and variants of the *GC* gene. Therefore, it is not clear if bioavailable or free 25(OH)D offer additional benefits compared to total 25(OH)D when estimating the magnitude of these associations. Our aim was to evaluate the association between 25(OH)D (total, free and bioavailable) with adiposity and metabolic traits. This was a cross-sectional study of 1904 subjects from the Health Workers Cohort Study from Mexico. Free and bioavailable 25(OH)D were calculated based on VDBP and albumin determinations, using a formula adjusted for the *GC* gene diplotypes. Adiposity and metabolic traits were measured with standardized procedures. Free and bioavailable 25(OH)D levels correlated with total 25(OH)D, r = 0.71 and 0.70, respectively (*p* < 0.001). Total, bioavailable and free 25(OH)D levels were negatively associated with the adiposity marker (visceral adiposity index) and metabolic traits (metabolic syndrome, type 2 diabetes, triglycerides, triglycerides/HDL-c ratio, and triglycerides/glucose index) in multivariate regression models (ORs = 0.73 to 0.96). Our findings suggest that free and bioavailable 25(OH)D do not offer additional advantages over total 25(OH)D regarding its association with adiposity and several metabolic traits in Mexican adults.

## 1. Introduction

Vitamin D is an essential micronutrient mainly involved in calcium metabolism and bone mineralization [1]. Vitamin D can be obtained from vitamin D-containing foods and supplements or synthesized in the skin cells from 7-dehydrocholesterol after sunlight exposure. Vitamin D is metabolized into 25-hydroxyvitamin D [25(OH)D] in the liver through the action of 25-hydroxylase (CYP2R1). In the kidneys, this metabolite is converted into its biologically active form, 1α,25-dihydroxyvitamin D [1α,25(OH)_2_D] by the 25-hydroxyvitamin D-1α-hydroxylase (CYP27B1) [2]. Total 25(OH)D has been widely accepted as the main indicator to determine the nutritional status of vitamin D among individuals [3]; however, different fractions of 25(OH)D exist. Between 85–90% of the total circulating 25(OH)D is bound to the vitamin D-binding protein (VDBP), nearly 15% is bound to albumin, and less than 1% is in a free form [4]. The 25(OH)D bound to the VDBP is biologically inactive, the fraction bound to albumin is considered biologically available, and the free form is recognized as the most active form according to the “free hormone hypothesis” [5,6]. The *GC* gene encodes VDBP and its isoforms significantly impact the affinity and bioavailability of vitamin D metabolites. The main genetic variants in *GC* are two single nucleotide polymorphisms (SNPs), the rs7041 and rs4588. Previous studies had investigated the effect of these SNPs on the affinity to vitamin D [7,8]. From these SNPs arise the three most common alleles of *GC* gene GC1f (rs7041-T/rs4588-C); GC1s (rs7041-G/rs4588-C), and GC2 (rs7041-T/rs4588-A), which differentially influence the concentration of VDBP and its binding affinity to 25(OH)D [8,9,10,11,12]. In a previous study in postmenopausal women participating in the Health Workers Cohort Study (HWCS), we observed that women carrying the GC2/2 and GC1f/2 genotypes had higher odds for presenting vitamin D deficiency compared to women carrying the GC1f/GC1s genotype, indicating the importance of genotype adjustment for the estimation of free and bioavailable 25(OH)D [13].

Based on the serum levels of 25(OH)D, vitamin D nutritional status has been classified as deficient (<20 ng/mL), insufficient (21–29 ng/mL), and sufficient (>30 ng/mL) [2]. Recent evidence has shown an association of vitamin D deficiency with several metabolic disturbances such as metabolic syndrome (MetS), high blood pressure (BP), type 2 diabetes (T2D), insulin resistance (IR), and obesity [14,15,16,17,18]. Diverse studies have suggested that vitamin D deficiency could promote adipogenesis and disturb the metabolism involved in energy homeostasis, such as leptin, adiponectin, and resistin [19,20,21]. However, these associations are still under debate. Some research has focused on total 25(OH)D without considering VDBP levels, which have proved to regulate free and total vitamin D metabolites related to numerous clinical conditions [22]. Nevertheless, the association of the different forms of Vitamin D with the above-described metabolic traits has been scarcely explored. The aim of this study was to evaluate the association of total, free, and bioavailable 25(OH)D with sociodemographic characteristics, adiposity markers, and metabolic traits in a cohort of Mexican-mestizo individuals.

## 2. Materials and Methods

### 2.1. Study Population

A cross-sectional analysis was conducted using data from adult participants in the HWCS. The HWCS is designed to investigate genetic, social, and environmental factors of chronic diseases. The design, selection criteria, and methods of the HWCS have been reported elsewhere [23]. A total of 2086 individuals (age range 5–92 years old) with serum samples were included from the second measurement period of the HWCS (from 2010 to 2012). For the present analysis, we excluded 85 individuals under 18 years old, 97 subjects with missing data regarding levels of serum 25(OH)D, VDBP, waist circumference, or genotypes of the *GC* gene, resulting in a final sample size of 1904. The study protocol was approved by the National Research and Ethics Committee of the Mexican Social Security Institute (IMSS, by its Spanish acronym) and the National Institute of Genomic Medicine (INMEGEN). All participants provided written informed consent.

### 2.2. Biochemical, Clinical and Anthropometric Measures 

Venous blood samples were collected after an 8-h fast. Total serum 25(OH)D was measured with the LIAISON^®^ 25OH Vitamin D Total Assay (Diasorin, Saluggia (VC), Italy) (intra- and inter-assay variation coefficients were <10%) [24]. Serum VDBP was measured using the ELISA technique with a commercial kit (Quantikine ELISA kit (R&D Systems, Minneapolis, MN, USA, Cat No. DVDBP0B). This kit employs quantitative sandwich enzyme immunoassay using a monoclonal antibody (intra and inter-assay coefficient of variation, <7%) [25].

Albumin was measured by a colorimetric method (bromcresol green) using UniCel^®^ DxC 600/800 System(s) and Synchron^®^ Systems Multi Calibrator, Beckman Coulter (intra and inter-assay coefficient of variation, <4.5%) [26]. Glucose levels were assessed with the oxidized glucose method. Triglycerides (TG) were determined with a colorimetric method after enzymatic hydrolysis with lipases technique, and high-density lipoprotein cholesterol (HDL-c) was measured by eliminating chylomicron and subsequent catalase. These laboratory procedures were standardized according to the International Federation of Clinical Chemistry and Laboratory Medicine [27]. The Homeostatic Model Assessment for insulin resistance (HOMA-IR) was determined with the formula: HOMA-IR = (insulin μU/mL × glucose mmol/L)/22.5 [28].

Weight and height were measured using a calibrated electronic TANITA scale and a conventional stadiometer, respectively, with methods that have been described previously [23]. Waist circumference was measured with a steel measuring tape at the high point of the iliac crest at the end of normal expiration to the nearest 0.1 cm. BP was measured using an electronic digital BP monitor. BP was measured twice at 5-min intervals, and the mean of the two readings was recorded. All clinical measurements were conducted by trained staff using standardized methods [23].

### 2.3. Genotyping of GC Gene Variants

DNA was extracted from peripheral blood using the QIAamp DNA Blood Mini Kit according to the manufacturer’s instructions. Two *GC* SNPs (rs4588 and rs7041) were genotyped. Genotyping was performed using predesigned TaqMan SNP Genotyping assays (Applied Biosystems, Massachusetts, MA, USA) in a QuantStudio 7 Flex Real-Time PCR system (Applied Biosystems, Massachusetts, United States). An automatic variant call was done by the SDS software version 2.2.1. The frequencies of rs7041 TT, TG, and GG genotypes were 27.7%, 49.9%, and 22.3%, respectively. The genotype frequencies of rs4588 SNP were 62.2% for CC, 35.0% for CA, and 2.7% for AA. The frequencies of the haplotypes (rs7041/rs4588) were homozygous GC1S/1S (22.32%), 1F/1F (10.4%) and 2/2 (2.7%) and heterozygous 1F/1S (29.6%), 1F/2 (14.7%) and 1S/2 (20.3%).

### 2.4. Estimation of Free and Bioavailable 25(OH)D

We estimated free 25(OH)D (pg/mL) and bioavailable 25(OH)D (ng/mL) with equations previously described and adapted for free testosterone estimation [4,29,30]. For these equations we used total 25(OH)D (ng/mL), serum VDBP concentrations (μg/mL), and serum albumin concentrations (g/dL) (Supplementary Material S1). SNP-adjusted free and bioavailable 25(OH)D was calculated by placing a haplotype-specific affinity constant reported in a previous study [7]. To assign binding coefficients for the specific *GC* phenotypes, we used the genotypes of the rs7041 and rs4588 variants. Diplotype-corrected free 25(OH)D concentration was calculated based on the affinity constants for Gc-1S, Gc-1F, and Gc-2. The binding coefficients were calculated as the mean of the corresponding haplotypes present in each individual according to Johnsen et al. [12]. The diplotype-corrected affinity constants were as follows: 1S/1S = 6 × 10^8^, 1S/1F = 4.8 × 10^8^, 1S/2 = 8.6 × 10^8^, 1F/1F = 3.6 × 10^8^, 1F/2 = 7.4 × 10^8^, 2/2 = 11.2 × 10^8^ [12].

### 2.5. Outcome Measures

Each participant’s body mass index (BMI) was calculated as weight in kg/height in m^2^ and the World Health Organization (WHO) criteria were used to determine overweight or obesity status. Body fat proportion was determined by dual X-ray absorptiometry (DXA; Lunar DPX-GE, Lunar Radiation, software version 1.35, fast scan mode) (Intra- and inter- assay variation coefficients were within usual operational standards and were lower than 1.5%) classified by tertiles [23].

MetS was defined as having three or more of the following criteria, based on the modified National Cholesterol Education Program (NCEP)—Adult Treatment Panel (ATP) III: (1) waist circumference ≥102 cm in males and ≥88 cm in females, (2) elevated TG ≥150 mg/dL or medical treatment for elevated TG, (3) reduced HDL-c <40 mg/dL in males and <50 mg/dL in females, (4) elevated systolic BP ≥130 mmHg and/or diastolic BP ≥85 mmHg or current use of antihypertensive drugs, (5) elevated fasting glucose ≥100 mg/dL or medical diagnosis of T2D [31].

Impaired glucose tolerance was defined as having fasting glucose ≥100 to <126 mg/dL. T2D was defined with one of the following three criteria: self-report of physician-diagnosed T2D, use of hypoglycemic medication, or fasting glucose ≥126 mg/dL [32].

Fasting TG and glucose index (TyG index) was calculated as the ln [fasting TG (mg/dL) × fasting plasma glucose (mg/dL)/2]. This index has been demonstrated to be an adequate and affordable surrogate for determining insulin resistance when insulin measurement is unavailable [33]. The absolute TG levels (mg/dL) were divided by absolute HDL-c levels (mg/dL) to calculate the TG/HDL-c ratio. Previous studies have shown that the TG/HDL-c ratio can be a reliable marker of insulin resistance and glycemic control [34]. Visceral adiposity index (VAI) is a sex-specific index determined using a formula previously described by Amato et al. [35]. The TyG index, TG/HDL-c ratio, and VAI were classified based on tertiles. IR was defined as a HOMA-IR ≥3.2 [36].

### 2.6. Measurement of Other Covariates

Demographic data (age and sex), medication use and lifestyle factors (e.g., physical activity and smoking status) were obtained through a self-reported questionnaire [23]. Smoking status was classified as current, past, or never. We estimated dietary intake of vitamin D using a semi-quantitative food frequency questionnaire (FFQ), and we obtained the information on nutrient intake from a comprehensive database of food contents [23,37]. Vitamin D intake was classified by tertiles. Leisure-time physical activity was assessed through a validated physical activity questionnaire [38], and participants were classified as active if their physical activity was >150 min/week. The season of blood draw was categorized into spring (March, April, May), summer (June, July, August), autumn (September, October, November), and winter (December, January, February).

### 2.7. Statistical Analyses

Continuous data are presented as median and interquartile range and categorical variables as proportions. The differences between medians or proportions by sex were assessed using the Mann–Whitney and chi-square tests, respectively. Adjusted medians were derived from multivariable quantile regression models that included the following variables: age, sex, the season of blood collection, smoking status and, vitamin D intake. To evaluate the association between vitamin D (total, free and bioavailable) with adiposity markers and metabolic traits, we used binary and multinomial logistic regression models adjusted for covariates. Binary logistic regression models were applied for outcomes such as HOMA-IR, MetS, and its components. Multinomial logistic regressions were performed for BMI status, TyG index, TG/HDL-c ratio, and VAI. These models were adjusted for confounding factors such as sex, age groups, the season of blood draw, vitamin D consumption, leisure-time physical activity, and smoking status. Our models met the assumptions of linearity in the logit for any continuous independent variables [e.g., total, free and bioavailable 25(OH)D], absence of multicollinearity, goodness-of fit, specification model (no important variables were omitted), and lack of strongly influential outliers. Furthermore, we used quantile regression, modeling the association between different forms of Vitamin D and adiposity and metabolic traits as continuous variables. This nonparametric statistical method models the median of the outcome variables and any other percentile across their distribution without categorizing the variable. Finally, we evaluated the Spearman correlation between total 25(OH)D and bioavailable or free 25(OH)D. All *p*-values are two-tailed, and a *p* < 0.05 was considered significant. Statistical analyses were conducted using Stata 14.0 (StataCorp, College Station, TX, USA).

## 3. Results

### 3.1. Demographic Characteristics of the Study Population

This study included 1904 individuals from the HWCS, of which 31% were men and 69% were women. Most of the measured parameters showed significant differences between men and women (*p* < 0.05); however, BMI, T2D, HOMA-IR, and Vitamin D intake did not show a statistical difference (*p* > 0.05). The vitamin D deficiency and levels of VDBP were higher in women than in men (Table 1).

### 3.2. Levels of Total, Free and Bioavailable 25-Hydroxyvitamin D According to Clinical Data

The median of total, free and bioavailable 25(OH)D was stratified by clinical characteristics (Table 2). Individuals with adiposity markers, such as obesity and body fat proportion (in the highest tertile), had significantly lower levels of total, free, and bioavailable 25(OH)D; whereas individuals with overweight only had lower levels of total and bioavailable 25(OH)D compared to individuals in the lowest category (*p* < 0.05). In addition, individuals with MetS and its components (abdominal obesity, hypertriglyceridemia, and elevated fasting plasma glucose) showed lower levels of total, free and bioavailable 25(OH)D compared to individuals without MetS (*p* < 0.05). We found significant differences of total and bioavailable 25(OH)D in individuals with low levels of HDL-c (*p* < 0.05) but not with free 25(OH)D. The medians of total, free and bioavailable 25(OH)D were lower in individuals with T2D. In contrast, individuals with IR only had significantly lower levels of total and bioavailable 25(OH)D compared with participants without T2D or IR (*p* < 0.05). Individuals with novels markers for metabolic disorders, such as TyG index, TG/HDL-c ratio and, VAI (in the highest tertile), had significantly lower levels of total, free and bioavailable 25(OH)D (*p* < 0.001).

Furthermore, the median of total, free and bioavailable 25(OH)D was stratified by demographic characteristics (Supplementary Table S1). We observed that the adjusted medians of total, free and bioavailable 25(OH)D were lower in women than men (*p* < 0.001). The highest median of total 25(OH)D was found among individuals in the fifth decade of life (22.1 ng/mL, *p* < 0.001). In contrast, the highest median of free 25(OH)D was found among individuals in the sixth decade of life (6.7 pg/mL, *p* < 0.05). The bioavailable 25(OH)D was not significantly different between age groups. Participants whose blood was collected during spring had the highest median of the total, free and bioavailable 25(OH)D (22.0 ng/mL, 6.9 ng/mL, and 2.7 ng/mL, respectively; *p* < 0.001), compared to samples obtained in winter. Individuals with active leisure-time physical activity had significantly lower levels of free and bioavailable 25(OH)D compared with inactive individuals (*p* < 0.005). On the other hand, none of the 25(OH)D parameters was associated with smoking status.

Total, free, and bioavailable 25(OH)D correlated negatively with BMI, body fat proportion, TG, TyG index, TG/HDL ratio, and VAI (Supplementary Table S2).

### 3.3. Association between Vitamin D Levels, Adiposity Markers and Metabolic Traits

We observed that total, free and bioavailable 25(OH)D levels were associated as a protective factor with the adiposity marker: medium and high VAI (OR = 0.75–0.96) and metabolic traits: MetS (OR = 0.88–0.96), T2D (OR = 0.86–0.95), high TG (OR = 0.75–0.93), TyG index (OR = 0.73–0.95) and TG/HDL-c ratio (OR = 0.73–0.96), even after adjusting for potential confounding factors.

Total and bioavailable 25(OH)D had a significant association with obesity (OR = 0.95; 95% CI: 0.93–0.97 and OR = 0.89; 95% CI: 0.86–0.99, respectively) and high body fat proportion (OR = 0.96; 95% CI: 0.93–0.98 and OR = 0.86; 95% CI: 0.75–0.98, respectively).

In addition, we observed a significant association between overweight (OR = 0.97; 95% CI: 0.95,0.99), high waist circumference (OR = 0.96; 95% CI: 0.95,0.98), impaired glucose (OR = 0.98; 95% CI: 0.96,0.99), low HDL-c (OR = 0.98; 95% CI: 0.97,0.99) and HOMA-IR (OR = 0.96; 95% CI: 0.94,0.98) only with total 25(OH)D (Table 3). We further analyzed these associations in women and men separately, and the results were almost identical in both sexes (Supplementary Table S3).

The quantile regression identified a negative association between the different forms of vitamin D (total, free and bioavailable) and the adiposity indicators (BMI, body fat proportion, and waist circumference) at the 50th quantile. Furthermore, we observed a negative association between the different forms of vitamin D (total, free and bioavailable) and TG, TyG index, TG/HDL ratio, and VAI at the 25th, 50th, and 75th percentiles (Supplementary Table S4).

**Figure 1:**
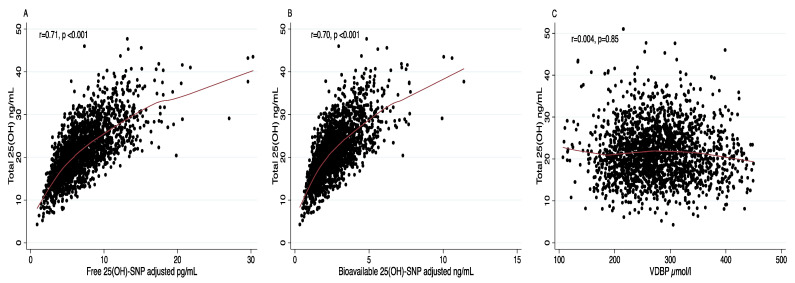
Scatter plot representation of the correlation between total 25(OH)D with free 25(OH)D (**A**), bioavailable 25(OH)D (**B**) and VDBP (**C**) in 1904 subjects from the Health Workers Cohort Study.

Additionally, we analyzed the correlation between total, free and bioavailable 25(OH)D. Positive and significant correlations were observed in the overall population between total and free 25(OH)D (r = 0.71, *p* < 0.001) (Figure 1A), as well as with bioavailable 25(OH)D (r = 0.70, *p* < 0.001) (Figure 1B). The correlations of total 25(OH)D with free and bioavailable 25(OH)D were expected since the free and bioavailable fractions were mathematically calculated from total 25(OH)D levels. On the contrary, there was no correlation between VDBP and total 25(OH)D (Figure 1C). These results were similar among men (Supplementary Figure S1A–C) and women (Supplementary Figure S2A–C).

## 4. Discussion

Total 25(OH)D has been accepted as the uniform parameter to determine the nutritional status of vitamin D; however, the bioavailable and free fractions are responsible for its biological effects. Here, we analyzed the association between the different 25(OH)D fractions with adiposity and metabolic traits in a cohort of Mexican-mestizo individuals.

Free and bioavailable 25(OH)D were calculated using the modified Vermeulen method for free testosterone estimation [39]. This method gives separate measurements of free and bioavailable 25(OH)D, in contrast to the Bikle et al., a method that only gives the free 25(OH)D [39,40]. However, studies have shown that the results of these two methods are significantly correlated since they are all calculated from the total 25(OH)D [40].

There is a general belief that serum (total) 25(OH)D is the best biochemical marker of the vitamin D status [41]. More recently, there have been several studies suggesting that unbound (free or bioavailable) 25(OH)D concentrations may be a better marker for several outcomes (bone, PTH, or other non-skeletal effects) than total 25(OH)D. Whether free or bioavailable 25(OH)D would be better markers than total 25(OH)D is so far unclear. Results of this study suggest that total and calculated free or bioavailable 25(OH)D levels are associated with adiposity markers and metabolic traits in our population; however, free or bioavailable 25(OH)D only modestly differ from total 25(OH)D for specific markers. Thus, our data do not support the notion that free or bioavailable 25(OH)D measures offer additional advantages over total 25(OH)D to evaluate the association between vitamin D status, adiposity markers, and metabolic traits in our population. To the best of our knowledge, this is the first study analyzing this association in the Mexican population.

We observed a positive correlation between free and bioavailable 25(OH)D with total 25(OH)D, which has been reported by other studies [42,43]. Pelczyńska et al. reported a strong correlation between total 25(OH)D with free (r = 0.794, *p* < 0.001) and bioavailable 25(OH)D (r = 0.817, *p* < 0.001) [42]. Similar results were observed in the Nurses’ Health Study II for free and total 25(OH)D (r = 0.76, *p* < 0.001) [43]; however, Oleröd et al., found a moderate correlations (r = 0.67, *p* < 0.001) [44].

Several studies have shown an association between 25(OH)D deficiency and obesity as a risk factor [21,44]. Some potential mechanisms that might explain this association include: (1) a decreased exposure to sunlight, (2) trapping of vitamin D in adipose tissue, which makes it less available for its conversion into 1α,25(OH)_2_D and (3) a decreased expression of the 1-α hydroxylase and CYP2R [45,46,47]. Recent observations also suggest that this association may also be related to the pro-inflammatory state and circulating cytokines present in obesity and an increased volume of distribution into adipose tissue [22].

In other studies, the calculated concentrations of free and bioavailable 25(OH)D were lower in men and women with obesity than normal-weight individuals [48], regardless of the method used for these estimations [45,46]. Our study observed significant differences in total, free, and bioavailable 25(OH)D levels among individuals with obesity and VAI (a marker visceral adipose function) in the medium and high tertile. On the other hand, individuals with overweight only presented significant differences in levels of total and bioavailable 25(OH)D. These data on the group of individuals of the health workers are consistent with previous reports that include high BMIs individuals, patients with cirrhosis, nursing home residents, and patients with prediabetes [47] reported to have lower total and free 25(OH)D levels. In addition, in an elderly population, free and bioavailable 25(OH)D do not appear to be superior to total 25(OH)D in predicting indices of bone health [48].

We found that total, free, and bioavailable 25(OH)D were significantly lower in subjects with MetS than those without MetS. Similar results were observed by Pelczyńska et al. [42]. Several studies have documented a decrease of total 25(OH)D among individuals with MetS [49,50,51]; however, there are slightly inconsistent results in the literature [52,53]. Possible reasons for these discrepancies are residual confounding, inadequate statistical power, and not considering the VDBP levels.

On the other hand, we observed an association between total vitamin D and HOMA-IR (continuous and categorical); however, we did not observe significant associations with free and bioavailable 25(OH)D. Few studies have explored the associations between different forms of vitamin D and IR. In women with polycystic ovary syndrome, total and free 25(OH)D were related to markers of IR, but when adjusted for BMI, the association was no longer significant [54]. Lee et al. observed that free and total 25(OH)D were positively associated with tissue insulin sensitivity index and β-cell function among non-diabetic subjects. However, after adjustment for BMI, only free 25(OH)D was significantly associated with insulin secretion [55]. In our study, the associations between different forms of 25(OH)D (total, free and bioavailable) and T2D remained statistically significant after adjustment for BMI (0.95, CI 95% 0.93–0.97; 0.95, CI 95% 0.91–0.99; 0.88, CI 95% 0.78–0.99; respectively). Results similar were observed between total 25(OH)D and HOMA-IR (0.97, CI 95% 0.95–0.99).

We observed an association, as a protective factor, of total, free and bioavailable 25(OH)D with obesity marker (VAI) and metabolic traits (MetS, T2D, high TG, TyG index, and TG/HDL-c ratio), even after adjusting for potential confounding factors. The “free hormone hypothesis” purports that VDBP may act as a carrier and reservoir, prolonging the half-life of 25(OH)D and, at the same time, regulating its immediate bioavailability to target tissues. It has recently been suggested that the biological actions of vitamin D are prevented when it is bound to VDBP [30]. Therefore, bioavailable 25(OH)D is the fraction able to exert an effect on adiposity and metabolic traits [6]. However, genotypic variations of the *GC* gene may be associated with significant changes in binding affinity and/or serum concentrations of VDBP. In our study, we observed that by not considering the adjustment for the *GC* diplotypes, in the calculation of free and bioavailable 25(OH)D, the association between bioavailable 25(OH)D and the various outcomes was overestimated (Supplementary Tables S5 and S6). Bacha et al. reported that young individuals with low concentrations of free and bioavailable 25(OH)D had lower insulin sensitivity and higher levels of inflammatory biomarkers [56]. However, the free and bioavailable 25(OH)D calculation did not consider adjustment for *GC* diplotypes. Therefore, we consider that adjustment for genetic variants of *GC* is essential when exploring the role of free and bioavailable 25(OH)D on adiposity and metabolic traits, especially in populations such as ours, with mixed ethnicity.

The present study is the first to investigate the levels of serum bioavailable 25(OH)D and free 25(OH)D in the Mexican adult population. Similar to our findings, a previous study in healthy young women also revealed that determination of different forms of 25(OH)D does not offer additional advantages over total 25(OH)D [57].

This study has some limitations. First, most of the HWCS participants have an education level higher than the general population. Therefore, our sample is not representative of the entire Mexican population. However, the HWCS population can be considered representative of adults living in urban areas in Central Mexico. Second, serum 25(OH)D concentration varies by season; we use the blood collection month and physical activity proxy for sun exposure. Third, the free and bioavailable 25(OH)D were not measured directly. Unfortunately, the direct quantification of these metabolites is technically complicated by the small percentage of free and bioavailable 25(OH)D in serum (~0.03%), making it more challenging to measure. Fourth, our questionnaire does not include information on sun exposure (we use blood collection season as a proxy) or consumption of vitamin D supplements, which is not common among the Mexican population [58].

Some of the strengths of this study are the measurement of sun exposure, outcomes, and covariates using high-quality standard methods, reducing the probability of information bias. Further, *GC* SNPs rs4588 and rs7041 were genotyped, which allowed us to estimate the genotype-specific affinity constants. These constants were used in the association analysis of free and bioavailable 25(OH)D; this represents an innovative biological approach.

Our results may have important epidemiological and clinical implications in our population. It is important to notice that the measure of bioavailable or free 25(OH)D levels is more expensive and requires further validation and standardization than total 25(OH)D [59,60]. Furthermore, the genotyping of specific VDBP phenotypes to more correctly calculate the free and/or bioavailable 25(OH)D levels would further increase the analysis cost.

Although it has been reported that free and bioavailable fractions of 25(OH)D are strongly linked to significant biological effects; in our population, the associations of free and bioavailable 25(OH)D adjusted by the different VDBP binding coefficients showed results similar to those observed with total 25(OH)D.

## 5. Conclusions

The present study indicates that the determination of free and bioavailable 25(OH)D does not offer additional advantages over total 25(OH)D regarding its association with adiposity and metabolic traits in the Mexican population. Furthermore, our results add evidence supporting the association between vitamin D metabolites and metabolic traits. Further studies are needed to confirm whether our findings have broader implications.

## Figures and Tables

**Table 1:** Demographics of 1904 individuals who belong to the Health Workers Cohort Study.

Parameter	Men	Women	
	*n* = 582	*n* = 1322	*p* Value
Age (years) ^a^	46 (36–57)	54 (43–63)	<0.001
Leisure time physical activity (hour/week) ^a^	1.7 (0.4–5.0)	1.1 (0.2–3.5)	<0.001
Active (>150 min/week), %	36.6	28.4	0.008
Smoking status			
Current, %	21.0	8.6	<0.001
Past, %	39.0	22.5	<0.001
BMI (kg/m^2^) ^a^	26.5 (24.1–29.0)	26.8 (24.0–30.1)	0.150
Overweight, %	48.9	40.3	<0.001
Obesity, %	19.5	25.7	0.003
Body fat proportion ^a^	31.5 (27.7–34.7)	45.1 (40.7–49.1)	<0.001
Metabolic syndrome (ATP III definition) ^b^, %	45.9	55.5	<0.001
Waist circumference (cm) ^a^	96 (90–102)	92 (85–100)	<0.001
Systolic blood pressure (mmHg) ^a^	122 (113–131)	116 (106–129)	<0.001
Diastolic blood pressure (mmHg) ^a^	77 (70–84)	73 (66–79)	<0.001
Elevated blood pressure, %	43.0	37.0	0.010
Fasting glucose(mg/dL)	98 (92–107)	96 (90–104)	<0.001
Impaired glucose tolerance, %	33.1	24.8	<0.001
Type 2 Diabetes, %	15.0	14.3	0.680
HDL-c(mg/dL) ^a^	39 (34–46)	46 (39–54)	0.007
Triglycerides(mg/dL) ^a^	168 (118–247)	151 (109–199)	<0.001
HOMA-IR (>3.2) ^c^, %	35.1	32.4	0.370
TyG index ^a,d^	4.3 (2.8–6.7)	3.3 (2.1–4.8)	<0.001
TG/HDL-c ratio ^a^	9.0 (8.7–9.5)	8.9 (8.6–9.2)	<0.001
Visceral adiposity index ^a^	2.6 (1.7–4.0)	2.9 (1.8–4.1)	<0.001
Total 25(OH)D (ng/mL) ^a^	22.1 (18.3–26.9)	20.8 (16.7–24.8)	<0.001
Free 25(OH)D-SNP adjusted (pg/mL) ^a,e^	7.1 (5.2–9.5)	6.4 (4.6–8.6)	<0.001
Bioavailable 25(OH)D-SNP adjusted (ng/mL) ^a,e^	2.7 (2.0–3.7)	2.4 (1.7–3.2)	<0.001
Albumin (g/dL) ^a^	4.3 (4.1–4.5)	4.2 (4.0–4.4)	<0.001
Vitamin D deficiency (<20 ng/mL), %	36.3	44.6	0.0008
Vitamin D-binding protein (µmol/L) ^a^	263.8 (229.6–303.8)	275.3 (234.0–318.4)	0.003
Vitamin D intake (UI/day) ^a^	145.2 (75.8–259.3)	144.5 (85.0–248.6)	0.370
Alcohol(g/day) ^a^	2.7 (0.6–7.4)	0.6 (0.0–1.8)	<0.001

^a^ Median (P25-P75). ^b^ NCEP-ATP III: National Cholesterol Education Program-Adult Treatment Panel III. ^c^ Data in a subsample of 1264 individuals. ^d^ TyG: triglycerides and glucose index. ^e^ Serum free and bioavailable 25(OH)D SNP adjusted.

**Table 2:** Levels of total, free and bioavailable 25(OH)D by demographic and clinical characteristics from the Health Workers Cohort Study.

Parameter	Total 25(OH)D (ng/mL) Median (95% CI)	*p* Value	Free 25(OH)D- SNP Adjusted ^a^ (pg/mL) Median (95% CI)	*p* Value	Bioavailable 25-(OH)D-SNP Adjusted ^a^ (ng/mL) Median (95% CI)	*p* Value
BMI ^b^						
Normal	22.0 (21.4,22.6)	Ref.	6.8 (6.5,7.0)	Ref.	2.7 (2.5,2.8)	Ref.
Overweight	20.8 (20.3,21.4)	0.008	6.6 (6.4,6.9)	0.260	2.5 (2.4,2.6)	0.013
Obesity	20.4 (19.7,21.1)	0.001	6.3 (6.0,6.6)	0.023	2.3 (2.2,2.4)	<0.001
Body fat proportion ^c,d^						
Low	21.9 (21.4,22.7)	Ref.	7.1 (6.9,7.4)	Ref.	2.7 (2.6,2.9)	Ref.
Medium	21.2 (20.6,21.7)	0.220	6.4 (6.2,6.7)	0.180	2.5 (2.3,2.6)	0.130
High	20.2 (19.5,20.9)	0.005	6.1 (5.9,6.4)	0.038	2.3 (2.2,2.4)	0.015
Metabolic syndrome-ATP III ^c^						
No	22.0 (21.5,22.4)		6.9 (6.6,7.1)		2.6 (2.5,2.7)	
Yes	20.3 (19.8,20.7)	<0.001	6.3 (6.1,6.5)	0.001	2.4 (2.3,2.5)	<0.001
High waist circumference ^c^						
No	21.9 (21.4,22.4)		6.8 (6.6,7.1)		2.6 (2.5,2.7)	
Yes	20.4 (20.0,20.9)	<0.001	6.4 (6.2,6.6)	0.060	2.4 (2.3,2.5)	0.024
Elevated blood pressure ^c^						
No	21.3 (20.9,21.8)		6.7 (6.5,6.9)		2.6 (2.5,2.6)	
Yes	20.7 (20.1,21.2)	0.070	6.3 (6.1,6.6)	0.004	2.4 (2.3,2.5)	0.003
Type 2 diabetes ^c^						
No	21.6 (21.1,22.1)	Ref.	6.7 (6.5,6.9)	Ref.	2.5 (2.4,2.6)	Ref.
Impaired glucose tolerance	20.6 (20.3,21.3)	0.010	6.8 (6.5,7.1)	0.630	2.5 (2.4,2.7)	0.710
Yes	19.8 (18.9,20.7)	<0.001	6.1 (5.6,6.5)	0.020	2.3 (2.1,2.4)	0.025
HDL-c ^c^						
Normal	21.5 (21.0,22.1)	Ref.	6.8 (6.6,7.0)	Ref.	2.6 (2.5,2.7)	Ref.
Low	20.8 (20.4,21.3)	0.05	6.5 (6.3,6.7)	0.150	2.4 (2.3,2.5)	0.025
Triglycerides ^c^						
Normal	22.5 (22.1,23.0)	Ref.	7.1 (6.9,7.3)	Ref.	2.7 (2.6,2.8)	Ref.
High	20.0 (19.6,20.5)	<0.001	6.1 (5.9,6.3)	<0.001	2.3 (2.2,2.4)	<0.001
TyG index ^c,d,e^						
Low	22.9 (22.4,23.5)	Ref.	7.2 (6.9,7.4)	Ref.	2.7 (2.6,2.8)	Ref.
Medium	21.0 (20.5,21.6)	<0.001	6.6 (6.4,6.9)	0.001	2.5 (2.4,2.6)	0.004
High	19.5 (18.9,20.0)	<0.001	6.1 (5.8,6.4)	<0.001	2.3 (2.2,2.4)	<0.001
TG/HDL-c ratio ^c,d^						
Low	22.9 (22.3,23.5)	Ref.	7.2 (7.0,7.5)	Ref.	2.7 (2.6,2.8)	Ref.
Medium	20.8 (20.2,21.4)	<0.001	6.5 (6.3,6.8)	<0.001	2.5 (2.4,2.6)	0.001
High	19.9 (19.3,20.5)	<0.001	6.2 (5.9,6.4)	<0.001	2.3 (2.2,2.4)	<0.001
Visceral adiposity index (VAI) ^c,d^						
Low	22.8 (22.3,23.4)	Ref.	7.1 (6.9,7.4)	Ref.	2.7 (2.6,2.8)	Ref.
Medium	21.1 (20.5,21.6)	<0.001	6.7 (6.4,6.9)	0.050	2.5 (2.4,2.6)	0.045
High	19.7 (19.1,20.2)	<0.001	6.1 (5.8,6.3)	<0.001	2.3 (2.2,2.4)	<0.001
HOMA-IR (>3.2) ^c,f^						
No	21.4 (20.9,21.8)	0.002	6.5 (6.3,6.8)	0.080	2.5 (2.4,2.6)	0.010
Yes	20.0 (19.4,20.7)		6.3 (5.9,6.6)		2.3 (2.1,2.4)	

^a^ Serum-free and bioavailable 25(OH)D SNP haplotype adjusted considering the specific binding coefficients for each of the six possible phenotypes of VDBP. ^b^ Median adjusted for sex, age groups, season of serum sampling, vitamin D consumption (tertiles), leisure-time physical activity, smoking status, and BMI. ^c^ Median adjusted for sex, age groups, season of serum sampling, vitamin D consumption (tertiles), leisure-time physical activity, smoking status. ^d^ Low, medium, and high category defined by tertiles. ^e^ TyG: triglycerides and glucose index. ^f^ Data in a subsample of 1263 individuals.

**Table 3:** Association between total, free, and bioavailable 25(OH)D levels, adiposity markers, and metabolic traits in the Health Workers Cohort.

	25(OH)D (ng/mL)	*p* Value	Free 25(OH)D SNP Adjusted (pg/mL) ^a^	*p* Value	Bioavailable 25(OH)D SNP Adjusted ^a^ (ng/mL)	*p* Value
Outcome ^b^	OR (95% CI)		OR (95% CI)		OR (95% CI)	
BMI						
Normal	Ref.		Ref.		Ref.	
Overweight	0.97 (0.95,0.99)	0.001	0.98 (0.95,1.01)	0.20	0.93 (0.85,1.00)	0.07
Obesity	0.95 (0.93,0.97)	<0.001	0.69 (0.54,0.88)	0.24	0.89 (0.86,0.99)	0.031
Body fat proportion						
Low	Ref.		Ref.		Ref.	
Medium	0.98 (0.97,1.00)	0.09	0.98 (0.94,1.03)	0.45	0.93 (0.82,1.05)	0.23
High	0.96 (0.93,0.98)	0.001	0.96 (0.92,1.01)	0.15	0.86 (0.75,0.98)	0.022
Metabolic syndrome-ATP III						
No	Ref.		Ref.		Ref.	
Yes	0.95 (0.94,0.97)	<0.001	0.96 (0.93,0.99)	0.003	0.88 (0.81,0.95)	0.001
Metabolic syndrome-ATP III ^c^						
No	Ref.		Ref.		Ref.	
Yes	0.96 (0.95–0.98)	<0.001	0.95 (0.92–0.99)	0.006	0.89 (0.81–0.97)	0.007
High waist circumference						
No	Ref.		Ref.		Ref.	
Yes	0.96 (0.95,0.98)	<0.001	0.99 (0.96,1.02)	0.55	0.94 (0.86,1.01)	0.12
Type 2 diabetes						
No	Ref.		Ref.		Ref.	
Impaired glucose tolerance	0.98 (0.96,0.99)	0.025	0.99 (0.95,1.02)	0.41	0.98 (0.90,1.07)	0.63
Yes	0.94 (0.92,0.97)	<0.001	0.95 (0.90,0.99)	0.015	0.86 (0.77,0.97)	0.016
Type 2 diabetes ^c^						
No	Ref.		Ref.		Ref.	
Impaired glucose tolerance	0.99 (0.97–1.01)	0.154	0.99 (0.96–1.02)	0.497	0.99 (0.91–1.08)	0.882
Yes	0.95 (0.93–0.97)	<0.001	0.95 (0.91–0.99)	0.023	0.88 (0.78–0.99)	0.036
Elevated blood pressure						
No	Ref.		Ref.		Ref.	
Yes	0.98 (0.97,0.99)	0.042	0.96 (0.94,0.99)	0.044	0.93 (0.85,1.00)	0.060
Elevated blood pressure ^c^						
No	Ref.		Ref.		Ref.	
Yes	0.99 (0.98–1.01)	0.405	0.97 (0.94–1.00)	0.065	0.94 (0.86–1.02)	0.158
Low HDL-c						
No	Ref.		Ref.		Ref.	
Yes	0.98 (0.97,0.99)	0.015	0.99 (0.96,1.02)	0.440	0.94 (0.87,1.01)	0.09
Low HDL-c ^c^						
No	Ref.		Ref.		Ref.	
Yes	0.99 (0.97–1.00)	0.133	0.99 (0.96–1.02)	0.579	0.95 (0.88–1.02)	0.179
High triglycerides						
No	Ref.		Ref.		Ref.	
Yes	0.93 (0.92,0.95)	<0.001	0.89 (0.87,0.92)	<0.001	0.75 (0.70,0.82)	<0.001
High triglycerides ^c^						
No	Ref.		Ref.		Ref.	
Yes	0.94 (0.92–0.95)	<0.001	0.90 (0.87–0.92)	<0.001	0.76 (0.70–0.82)	<0.001
TyG index ^d,e^						
Low	Ref.		Ref.		Ref.	
Medium	0.95 (0.94,0.97)	<0.001	0.95 (0.92,0.98)	0.002	0.85 (0.79,0.94)	0.001
High	0.93 (0.91,0.95)	<0.001	0.88 (0.84,0.91)	<0.001	0.73 (0.66,0.80)	<0.001
TyG index ^c,d,e^						
Low	Ref.		Ref.		Ref.	
Medium	0.96 (0.94–0.98)	<0.001	0.95 (0.91–0.98)	0.002	0.86 (0.79–0.95)	0.002
High	0.91 (0.89–0.93)	<0.001	0.88 (0.84–0.91)	<0.001	0.73 (0.66–0.81)	<0.001
TG/HDL ratio ^d^						
Low	Ref.		Ref.		Ref.	
Medium	0.96 (0.94,0.98)	<0.001	0.94 (0.90,0.97)	<0.001	0.85 (0.75,0.90)	<0.001
High	0.91 (0.89,0.93)	<0.001	0.90 (0.86,0.93)	<0.001	0.74 (0.67,0.82)	<0.001
TG/HDL ratio ^b,d^						
Low	Ref.		Ref.		Ref.	
Medium	0.96 (0.94–0.97)	<0.001	0.93 (0.90–0.97)	<0.001	0.82 (0.75–0.90)	<0.001
High	0.58 (0.44–0.76)	<0.001	0.89 (0.86–0.93)	<0.001	0.75 (0.68–0.83)	<0.001
Visceral adiposity index (VAI) ^d^						
Low	Ref.		Ref.		Ref.	
Medium	0.96 (0.94,0.98)	<0.001	0.95 (0.92,0.99)	0.005	0.86 (0.79,0.94)	0.001
High	0.92 (0.90,0.94)	<0.001	0.90 (0.87,0.93)	<0.001	0.75 (0.68,0.83)	<0.001
Visceral adiposity index (VAI) ^c,d^						
Low	Ref.		Ref.		Ref.	
Medium	0.96 (0.94–0.98)	<0.001	0.95 (0.92–0.99)	0.006	0.87 (0.79–0.95)	0.002
High	0.93 (0.91–0.95)	<0.001	0.90 (0.95–0.93)	<0.001	0.76 (0.69–0.84)	<0.001
HOMA–IR (>3.2) ^f^						
No	Ref.		Ref.		Ref.	
Yes	0.96 (0.94,0.98)	<0.001	0.97 (0.94,1.01)	0.18	0.92 (0.83,1.01)	0.091
HOMA–IR (>3.2) ^c,f^						
No	Ref.		Ref.		Ref.	
Yes	0.97 (0.95–0.99)	0.006	0.98 (0.94–1.02)	0.307	0.94 (0.84–1.05)	0.294

^a^ Serum-free and bioavailable 25(OH)D SNP haplotype adjusted considering the specific binding coefficients for each of the six possible phenotypes of VDBP. ^b^ All models were adjusted for sex, age groups, the season of serum sampling, vitamin D consumption, leisure-time physical activity, and smoking status. ^c^ Models additionally adjusted by BMI categories. ^d^ Low, medium and high categories are defined by tertiles. ^e^ TyG: triglycerides and glucose index. ^f^ Data in a subsample of 1263 individuals.

## Data Availability

The data used to support the findings of this study are available from the corresponding author for anyone who requests it.

## Supplementary Materials

The following are available online at https://www.mdpi.com/article/10.3390/nu13103320/s1, Supplementary Material S1. Calculation of Free and Bioavailable 25-hydroxyvitamin D concentrations. Supplementary Table S1. Levels of total, free and bioavailable 25(OH)D by demographic characteristics from the Health Workers Cohort Study. Supplementary Table S2. Spearman correlations between Vitamin D levels (total, bioavailable and free), adiposity markers and metabolic traits in the Health Workers Cohort Study. Supplementary Table S3. Association between total, free and bioavailable 25(OH)D levels, adiposity markers and metabolic traits by sex in the Health Workers Cohort Study. Supplementary Table S4. Quantile regression results for the different percentiles of adiposity markers and metabolic traits in the Health Workers Cohort Study. Supplementary Table S5. Association between total, free and bioavailable 25(OH)D levels, adiposity markers and metabolic traits in the Health Workers Cohort Study. Supplementary Table S6. Quantile regression results for the different percentiles of adiposity markers and metabolic traits in the Health Workers Cohort Study. Figure S1. Scatter plot representation of the correlation between total 25(OH)D with free 25(OH)D (A), bioavailable 25(OH)D (B) and VDBP (C) among males from the Health Workers Cohort Study. Figure S2. Scatter plot representation of the correlation between total 25(OH)D with free 25(OH)D (A), bioavailable 25(OH)D (B) and VDBP (C) among females from the Health Workers Cohort Study.
