# Supplementary material for: Total, Bioavailable, and Free 25-Hydroxyvitamin D Equally Associate with Adiposity Markers and Metabolic Traits in Mexican Adults
Source: Nutrients. 2021 Sep 23;13(10):3320. doi: 10.3390/nu13103320 (PMC8539380; doi:10.3390/nu13103320)
Supplement: Supplementary file 1 [file nutrients-13-03320-s001.zip › nutrients-1330969-supplementary.pdf]

nutrients

MDPI

Supplementary Materials

Total, Bioavailable and Free 25-Hydroxyvitamin D Equally Associate with Adiposity Markers and Metabolic Traits in Mexican Adults

Berenice Rivera-Paredez [1], Alberto Hidalgo-Bravo [2], Guadalupe León-Reyes [3], Leith S. León-Maldonado [4], Arnoldo Aquino-Gálvez [5], Manuel Castillejos-López [5], Edgar Denova-Gutiérrez [6], Yvonne N. Flores [7,8,9], Jorge Salmerón [1], and Rafael Velázquez-Cruz [3,*]

Supplementary material S1. Calculation of Free and Bioavailable 25-hydroxyvitamin D concentrations

Vermeulen Method:

a) Free 25-hydroxyvitamin D

$$[D_{free}] = \frac{-b + \sqrt{b^2 - 4ac}}{2a}$$

b) Bioavailable 25(OH)D

$$[Bio\ D] = [D_{free}] + [D_{alb}] = (K_{alb} \times [Alb] + 1) \times [D_{free}]$$

Where:

$a = K_{DBP} \times K_{alb} \times [Alb] + K_{DBP}$
$b = K_{DBP} \times [Total\ DBP] - K_{DBP} \times [Total\ D] + K_{alb} \times [Alb] + 1$
$c = -[Total\ D]$
[Alb] = concentration of albumin
[Total D] = concentration of total 25-hydroxyvitamin D
[VDBP] = concentration of Vitamin D-binding protein
[D_{Alb}] = Albumin-bound 25-hydroxyvitamin D = Bioavailable 25(OH)D−Free 25(OH)D

Affinity constant between 25(OH)D and albumin: $K_{alb} = 6 \times 10^5$ M^{-1}
Affinity constant between 25(OH)D and VDBP:

- for individuals carrying the 1S/S1 diplotype, KVDBP = 6 x $10^8 M^{-1}$
- for individuals carrying the 1S/1F diplotype, KVDBP = 4.8 x 10^8 M^{-1}
- for individuals carrying the 1S/2 diplotype, KVDBP = 8.6 x10^8 M^{-1}
- for individuals carrying the 1F/1F diplotype, KVDBP = 3.6 x 10^8 M^{-1}
- for individuals carrying the 1F/2 diplotype, KVDBP = 7.4 x 10^8 M^{-1}
- for individuals carrying the 2/2 diplotype, KVDBP = 11.2 x 10^8 M^{-1}

*Affinity constants are taken from Arnaud *et al* and Johnsen *et al* (7,12).

Calculated concentration of free 25-hydroxyvitamin D was expressed in pg/mL
Calculated concentration of bioavailable 25-hydroxyvitamin D was expressed in ng/mL

Table S1. Levels of total, free and bioavailable 25(OH)D by demographic characteristics from the Health Workers Cohort Study.

Parameter	Total 25(OH)D (ng/mL) Median (95% CI)	*P*-value	Free 25(OH)D- SNP adjusted[a] (pg/mL) Median (95% CI)	*P*-value	Bioavailable 25-(OH)D- SNP adjusted[a] (ng/mL) Median (95% CI)	*P*-value
Sex						
Men	22.1(21.4,22.7)	0.001	7.1(6.8,7.4)	<0.001	2.7(2.6,2.8)	<0.001
Women	20.7(20.3,21.1)		6.3(6.2,6.5)		2.4(2.3,2.5)	
Age groups						
18-29 years	19.4(18.4,20.4)	Ref.	6.2(5.8,6.7)	Ref.	2.5(2.3,2.7)	Ref.
30-39 years	21.8(20.8,22.7)	0.001	6.7(6.2,7.1)	0.160	2.6(2.4,2.8)	0.250
40-49 years	21.1(20.4,21.9)	0.008	6.6(6.3,7.0)	0.150	2.5(2.3,2.6)	0.650
50-59 years	22.1(21.5,22.8)	<0.001	6.6(6.3,6.9)	0.030	2.5(2.4,2.6)	0.160
60-69 years	20.9(20.2,21.7)	0.027	6.7(6.4,7.1)	0.019	2.5(2.4,2.7)	0.110
>70 years	19.7(18.6,20.8)	0.650	6.4(5.9,6.9)	0.350	2.4(2.2,2.6)	0.900
Season of blood sampling						
Winter	18.4(17.6,19.2)	Ref.	5.4(5.0,5.8)	Ref.	2.0(1.9,2.2)	Ref.
Spring	22.0(21.6,22.5)	<0.001	6.9(6.7,7.2)	<0.001	2.7(2.6,2.8)	<0.001
Summer	21.1(20.4,21.9)	<0.001	6.7(6.3,7.0)	<0.001	2.4(2.3,2.6)	<0.001
Autumn	21.1(20.1,22.1)	<0.001	6.7(6.3,7.2)	<0.001	2.5(2.3,2.7)	<0.001
Leisure time physical activity						
Inactive	20.9(20.5,21.3)	Ref.	6.4(6.2,6.6)	Ref.	2.4(2.3,2.5)	Ref.
Active	21.5(20.9,22.2)	0.480	6.9(6.7,7.2)	0.007	2.7(2.5,2.8)	0.005
Smoking status						
Never	20.9(20.5,21.4)	Ref.	6.6(6.4,6.8)	Ref.	2.5(2.4,2.6)	Ref.
Past	21.4(20.8,22.1)	0.230	6.7(6.4,7.0)	0.630	2.5(2.4,2.7)	0.460
Current	21.4(20.5,22.4)	0.390	6.6(6.2,7.1)	0.550	2.5(2.3,2.6)	0.180

[a] Serum free and bioavailable 25(OH)D haplotype adjusted considering the specific binding coefficients for each one of the six possible phenotypes of VDBP. All medians were adjusted for sex, age groups, the season of serum sampling, vitamin D consumption (tertiles), leisure-time physical activity, smoking status, and BMI.

Table S2. Spearman correlations between Vitamin D levels (total, bioavailable and free), adiposity markers and metabolic traits in the Health Workers Cohort Study.

Outcome	Total 25(OH)D (ng/mL)		Free 25(OH)D-SNP adjusted [a] (pg/mL)		Bioavailable 25(OH)D-SNP adjusted [a] (ng/mL)	
	rho	*P*-value	rho	*P*-value	rho	*P*-value
BMI	−0.107	<0.001	−0.039	0.09	−0.071	0.002
Body fat proportion	−0.140	<0.001	−0.119	<0.001	−0.150	<0.001
Waist circumference	−0.092	<0.001	−0.002	0.94	−0.028	0.22
Glucose levels	−0.085	0.0002	−0.026	0.25	−0.036	0.12
Systolic blood pressure	−0.051	0.02	0.001	0.95	−0.006	0.80
Diastolic blood pressure	0.005	0.81	0.010	0.60	0.006	0.81
HDL-c levels	0.012	0.60	−0.019	0.42	−0.008	0.72
Triglycerides levels	−0.205	<0.001	−0.166	<0.001	−0.159	<0.001
TyG index[b]	−0.213	<0.001	−0.157	<0.001	−0.154	<0.001
TG/HDL ratio	−0.166	<0.001	−0.124	<0.001	−0.123	<0.001
Visceral Adiposity Index (VAI)	−0.205	<0.001	−0.151	<0.001	−0.158	<0.001
Insulin levels	−0.084	0.003	−0.040	0.16	−0.052	0.06
HOMA−IR levels[c]	−0.093	0.0008	−0.041	0.15	−0.054	0.05

Table S3. Association between total, free and bioavailable 25(OH)D levels, adiposity markers and metabolic traits by sex in the Health Workers Cohort.

**Outcome** [b]	**Women**			**Men**		
	**25(OH)D (ng/mL)**	**Free 25(OH)D SNP adjusted** [a] **(pg/mL)**	**Bioavailable 25(OH)D SNP adjusted** [a] **(ng/mL)**	**25(OH)D (ng/mL)**	**Free 25(OH)D SNP adjusted** [a] **(pg/mL)**	**Bioavailable 25(OH)D SNP adjusted** [a] **(ng/mL)**
	**OR (95% CI)**	**OR (95% CI)**	**OR (95% CI)**	**OR (95% CI)**	**OR (95% CI)**	**OR (95% CI)**
BMI						
Normal	Ref.	Ref.	Ref.	Ref.	Ref.	Ref.
Overweight	**0.98* (0.95,0.99)**	0.99 (0.95,1.03)	0.94 (0.85,1.05)	**0.95* (0.93,0.98)**	0.96 (0.91,1.01)	0.88 (0.76,1.02)
Obesity	**0.96* (0.93,0.98)**	0.97 (0.93,1.02)	**0.87* (0.77,0.99)**	**0.92* (0.88,0.96)**	0.98 (0.91,1.05)	0.90 (0.74,1.08)
Body fat proportion						
Low	Ref.	Ref.	Ref.	Ref.	Ref.	Ref.
Medium	**0.97* (0.94,0.99)**	0.96 (0.92,1.00)	**0.88* (0.78,0.99)**	**0.96* (0.93,0.99)**	0.99 (0.94,1.06)	0.95 (0.81,1.12)
High	**0.96* (0.94,0.98)**	0.96 (0.91,1.00)	**0.85* (0.74,0.96)**	**0.91* (0.88,0.95)**	0.94 (0.87,1.00)	**0.81* (0.68,0.96)**
Metabolic syndrome− ATP III						
No	Ref.	Ref.	Ref.	Ref.	Ref.	Ref.
Yes	**0.96* (0.94,0.98)**	**0.95* (0.92,0.99)**	**0.87* (0.79,0.96)**	**0.94* (0.92,0.97)**	0.96 (0.91,1.01)	0.86 (0.76,1.00)
Metabolic syndrome− ATP III [c]						
No	Ref.	Ref.	Ref.	Ref.	Ref.	Ref.
Yes	**0.97* (0.95,0.99)**	**0.96* (0.92,0.99)**	**0.89* (0.80,0.99)**	**0.96* (0.93,0.99)**	0.96 (0.90,1.01)	0.88 (0.76,1.02)
High waist circumference						
No	Ref.	Ref.	Ref.	Ref.	Ref.	Ref.
Yes	0.99 (0.96,1.02)	1.00 (0.95,1.06)	0.99 (0.87,1.14)	0.96 (0.92,1.00)	0.98 (0.91,1.06)	0.92 (0.75,1.11)
Type 2 diabetes						
No	Ref.	Ref.	Ref.	Ref.	Ref.	Ref.

Impaired glucose tolerance	**0.97*** **(0.95,0.99)**	0.98 (0.94,1.02)	0.97 (0.87,1.08)	0.99 (0.96,1.02)	1.00 (0.95,1.06)	1.01 (0.87,1.16)
Yes	**0.94*** **(0.91,0.97)**	**0.92*** **(0.87,0.98)**	**0.80*** **(0.69,0.93)**	**0.95*** **(0.91,0.99)**	1.00 (0.92,1.08)	1.00 (0.81,1.22)
Type 2 diabetes [c]						
No	Ref.	Ref.	Ref.	Ref.	Ref.	Ref.
Impaired glucose tolerance	0.98 (0.99,1.00)	0.98 (0.94,1.02)	0.98 (0.89,1.10)	1.00 (0.97,1.03)	1.01 (0.96,1.07)	1.03 (0.89,1.19)
Yes	**0.94*** **(0.92,0.97)**	**0.92*** **(0.87,0.98)**	**0.82*** **(0.70,0.95)**	0.96 (0.92,1.01)	1.00 (0.93,1.09)	1.02 (0.83,1.26)
Elevated blood pressure						
No	Ref.	Ref.	Ref.	Ref.	Ref.	Ref.
Yes	0.99 (0.97,1.01)	0.97 (0.93,1.01)	0.93 (0.84,1.04)	0.98 (0.95,1.00)	0.96 (0.91,1.02)	0.92 (0.80,1.06)
Elevated blood pressure[c]						
No	Ref.	Ref.	Ref.	Ref.	Ref.	Ref.
Yes	1.00 (0.98,1.02)	0.98 (0.94,1.02)	0.95 (0.85,1.06)	0.99 (0.96,1.02)	0.97 (0.92,1.02)	0.94 (0.81,1.08)
Low HDL−c						
No	Ref.	Ref.	Ref.	Ref.	Ref.	Ref.
Yes	**0.98*** **(0.96,0.99)**	0.99 (0.96,1.03)	0.95 (0.87,1.05)	0.99 (0.96,1.01)	0.98 (0.93,1.03)	0.91 (0.80,1.03)
Low HDL−c[c]						
No	Ref.	Ref.	Ref.	Ref.	Ref.	Ref.
Yes	0.98 (0.96,1.00)	1.00 (0.96,1.04)	0.97 (0.88,1.07)	0.99 (0.97,1.02)	0.98 (0.93,1.03)	0.92 (0.81,1.05)
High triglycerides						
No	Ref.	Ref.	Ref.	Ref.	Ref.	Ref.
Yes	**0.94*** **(0.92,0.96)**	**0.90*** **(0.87,0.93)**	**0.76*** **(0.69,0.84)**	**0.92*** **(0.90,0.95)**	**0.89*** **(0.84,0.94)**	**0.73*** **(0.64,0.84)**
High triglycerides[c]						
No	Ref.	Ref.	Ref.	Ref.	Ref.	Ref.
Yes	**0.94***	**0.90***	**0.77***	**0.93***	**0.89***	**0.75***

	**(0.92,0.96)**	**(0.87,0.93)**	**(0.70,0.85)**	**(0.90,0.96)**	**(0.84,0.94)**	**(0.65,0.86)**
TyG index [d,e]						
Low	Ref.	Ref.	Ref.	Ref.	Ref.	Ref.
Medium	**0.94*<br>(0.91,0.96)**	**0.94*<br>(0.90,0.98)**	**0.83*<br>(0.74,0.93)**	**0.96*<br>(0.93,0.99)**	**0.94*<br>(0.88,0.99)**	**0.83*<br>(0.71,0.97)**
High	**0.95*<br>(0.93,0.97)**	**0.92*<br>(0.88,0.96)**	**0.79*<br>(0.70,0.88)**	**0.90*<br>(0.87,0.94)**	**0.86*<br>(0.81,0.93)**	**0.69*<br>(0.58,0.82)**
TyG index [c,d,e]						
Low	Ref.	Ref.	Ref.	Ref.	Ref.	Ref.
Medium	**0.96*<br>(0.94,0.98)**	**0.94*<br>(0.90,0.98)**	**0.84*<br>(0.75,0.94)**	**0.97*<br>(0.94,0.99)**	**0.93*<br>(0.88,0.99)**	**0.83*<br>(0.71,0.97)**
High	**0.94*<br>(0.92,0.96)**	**0.92*<br>(0.88,0.96)**	**0.79*<br>(0.70,0.90)**	**0.91*<br>(0.88,0.95)**	**0.87*<br>(0.81,0.93)**	**0.70*<br>(0.59,0.84)**
TG/HDL ratio [d]						
Low	Ref.	Ref.	Ref.	Ref.	Ref.	Ref.
Medium	**0.96*<br>(0.94,0.98)**	**0.94*<br>(0.90,0.98)**	**0.84*<br>(0.75,0.93)**	**0.96*<br>(0.93,0.99)**	0.97<br>(0.91,1.03)	**0.91*<br>(0.78,1.06)**
High	**0.91*<br>(0.89,0.94)**	**0.88*<br>(0.84,0.92)**	**0.73*<br>(0.65,0.82)**	**0.90*<br>(0.86,0.93)**	**0.86*<br>(0.80,0.92)**	**0.68*<br>(0.57,0,81)**
TG/HDL ratio [b,d]						
Low	Ref.	Ref.	Ref.	Ref.	Ref.	Ref.
Medium	**0.96*<br>(0.94,0.99)**	**0.94*<br>(0.90,0.98)**	**0.84*<br>(0.75,0.95)**	**0.97*<br>(0.94,0.99)**	0.97<br>(0.91,1.03)	0.91<br>(0.78,1.07)
High	**0.92*<br>(0.89,0.94)**	**0.88*<br>(0.84,0.92)**	**0.74*<br>(0.65,0.84)**	**0.91*<br>(0.87,0.94)**	**0.86*<br>(0.80,0.92)**	**0.69*<br>(0.58,0.83)**
Visceral Adiposity Index (VAI) [d]						
Low	Ref.	Ref.	Ref.	Ref.	Ref.	Ref.
Medium	**0.96*<br>(0.94,0.98)**	0.96<br>(0.92,1.00)	**0.89*<br>(0.79,0.99)**	**0.97*<br>(0.94,0.99)**	0.97<br>(0.91,1.03)	0.89<br>(0.77,1.04)
High	**0.94*<br>(0.91,0.96)**	**0.92*<br>(0.88,0.96)**	**0.79*<br>(0.70,0.89)**	**0.90*<br>(0.87,0.93)**	**0.87*<br>(0.81,0.93)**	**0.69*<br>(0.58,0.82)**
Visceral Adiposity Index (VAI) [c,d]						
Low	Ref.	Ref.	Ref.	Ref.	Ref.	Ref.

Medium	**0.96*** **(0.94,0.98)**	0.96 (0.92,1.01)	0.89 (0.80,1.00)	0.97 (0.94,1.01)	0.97 (0.91,1.03)	0.90 (0.76,1.05)
High	**0.94*** **(0.92,0.96)**	**0.92*** **(0.88,0.96)**	**0.80*** **(0.71,0.90)**	**0.91*** **(0.88,0.95)**	**0.87*** **(0.81,0.93)**	***0.70**** ***(0.59,0.84)***
HOMA−IR (>3.2) [f]						
No	Ref.	Ref.	Ref.	Ref.	Ref.	Ref.
Yes	**0.96*** **(0.93,0.98)**	0.96 (0.91,1.00)	**0.87*** **(0.77,0.99)**	0.97 (0.93,1.01)	1.02 (0.94,1.10)	1.02 (0.83,1.24)
HOMA−IR (>3.2) [c,f]						
No	Ref.	Ref.	Ref.	Ref.	Ref.	Ref.
Yes	**0.96*** **(0.93,0.99)**	0.96 (0.91,1.01)	0.89 (0.78,1.02)	0.98 (0.94,1.03)	1.03 (0.94,1.13)	1.08 (0.85,1.37)

[a] Serum free and bioavailable 25(OH)D haplotype adjusted considering the specific binding coefficients for each one of the six possible phenotypes of VDBP. [b]All models were adjusted for sex, age groups, the season of serum sampling, vitamin D consumption, leisure−time physical activity and smoking status. [c]Models additionally adjusted by BMI categories. [d] Low, medium and high category defined by tertiles. [e] TyG: triglycerides and glucose index. [f] Data in a subsample of 1,263 individuals. *P value <0.05.

Table S4. Quantile regression results for the different percentiles of adiposity markers and metabolic traits in the Health Workers Cohort.

**Outcome [b]**	**Total 25(OH)D (ng/mL)**			**Free 25(OH)D SNP adjusted [a] (pg/mL)**			**Bioavailable 25(OH)D SNP adjusted [a] (ng/mL)**		
	**Coefficient (95% CI)**			**Coefficient (95% CI)**			**Coefficient (95% CI)**		
	**25th**	**50th**	**75th**	**25th**	**50th**	**75th**	**25th**	**50th**	**75th**
BMI	**−0.09 ***<br>**(−0.13,−0.05)**	**−0.09***<br>**(−0.13,−0.06)**	**−0.12***<br>**(−0.18,−0.07)**	−0.04<br>(−0.11,0.03)	**−0.10***<br>**(−0.17,−0.03)**	−0.12<br>(−0.11,−0.09)	−0.16<br>(−0.33,0.02)	**−0.28***<br>**(−0.46,−0.09)**	−0.22<br>(−0.47,0.03)
Body fat proportion	**−0.19***<br>**(−0.26,−0.12)**	**−0.15***<br>**(−0.20,−0.10)**	**−0.12***<br>**(−0.18,−0.06)**	**−0.17***<br>**(−0.31,−0.04)**	**−0.14***<br>**(−0.24,−0.04)**	−0.07<br>(−0.19,0.05)	**−0.56***<br>**(−0.91,−0.22)**	**−0.46***<br>**(−0.72,−0.21)**	**−0.31***<br>**(−0.60,−0.01)**
Waist circumference	**−0.20***<br>**(−0.30,−0.10)**	**−0.26***<br>**(−0.34,−0.17)**	**−0.29***<br>**(−0.40,−0.17)**	−0.06<br>(−0.25,0.12)	−0.15<br>(−0.33,0.02)	−0.04<br>(−0.26,0.17)	−0.24<br>(−0.73,0.24)	**−0.54***<br>**(−0.99,−0.10)**	−0.27<br>(−0.82,0.29)
Glucose	**−0.11***<br>**(−0.20,−0.03)**	**−0.18***<br>**(−0.27,−0.09)**	**−0.25***<br>**(−0.43,−0.07)**	−0.11<br>(−0.28,0.05)	−0.14<br>(−0.33,0.05)	−0.19<br>(−0.55,0.17)	−0.24<br>(−0.67,0.19)	−0.22<br>(−0.71,0.27)	−0.56<br>(−1.45,0.40)
Glucose [c]	−0.03<br>(−0.11,0.05)	**−0.11***<br>**(−0.21,−0.02)**	**−0.22***<br>**(−0.40,−0.03)**	0.03<br>(−0.11,0.16)	−0.10<br>(−0.27,0.08)	−0.16<br>(−0.52,0.19)	0.21<br>(−0.14,0.56)	−0.18<br>(−0.65,0.29)	−0.35<br>(−1.29,0.59)
Diastolic blood pressure	−0.05<br>(−0.14,0.04)	−0.007<br>(−0.10,0.08)	−0.10<br>(−0.20,0.01)	−0.10<br>(−0.28,0.08)	−0.06<br>(−0.24,0.11)	−0.11<br>(−0.31,0.08)	−0.38<br>(−0.84,0.07)	−0.16<br>(−0.62,0.31)	−0.26<br>(−0.78,0.25)
Diastolic blood pressure [c]	0.0<br>(−0.09,0.09)	0.04<br>(−0.04,0.13)	−0.06<br>(−0.16,0.04)	−0.12<br>(−0.29,0.06)	−0.10<br>(−0.27,0.08)	−0.11<br>(−0.29,0.08)	−0.27<br>(−0.72,0.18)	−0.19<br>(−0.64,0.25)	−0.23<br>(−0.71,0.25)
Systolic blood pressure	−0.08<br>(−0.20,0.05)	**−0.16***<br>**(−0.30,−0.03)**	**−0.23***<br>**(−0.38,−0.09)**	−0.05<br>(−0.29,0.18)	−0.05<br>(−0.30,0.18)	**−0.35***<br>**(−0.62,−0.08)**	−0.14<br>(−0.76,0.47)	−0.54<br>(−1.20,0.13)	**−0.91***<br>**(−1.61,−0.20)**
Systolic blood pressure [c]	−0.04<br>(−0.16,0.08)	−0.12<br>(−0.25,0.005)	**−0.16***<br>**(−0.31,−0.01)**	−0.08<br>(−0.30,0.15)	−0.18<br>(−0.42,0.06)	−0.27<br>(−0.55,0.02)	−0.16<br>(−0.74,0.43)	−0.39<br>(−1.02,0.24)	−0.50<br>(−1.2,0.25)
HDL−c	0.03<br>(−0.05,0.11)	0.08<br>(−0.01,0.17)	0.18*<br>(0.07,0.29)	0.01<br>(−0.16,0.17)	0.01<br>(−0.16,0.19)	0.07<br>(−0.15,0.28)	0.18<br>(−0.24,0.60)	0.34<br>(−0.07,0.83)	0.32<br>(−0.25,0.90)
HDL−c [c]	0.02<br>(−0.06,0.11)	0.03<br>(−0.07,0.13)	0.14*<br>(0.03,0.26)	0.04<br>(−0.12,0.19)	−0.03<br>(−0.22,0.16)	0.07<br>(−0.15,0.30)	0.21<br>(−0.21,0.62)	0.08<br>(−0.40,0.57)	0.38<br>(−0.19.0.95)
Triglycerides	**−1.82***<br>**(−2.38,−1.26)**	**−2.54***<br>**(−3.15,−1.93)**	**−4.00***<br>**(−5.11,−2.84)**	**−2.75***<br>**(−3.86,−1.64)**	**−3.84***<br>**(−5.01,−2.67)**	**−5.38***<br>**(−7.46,−3.30)**	**−7.29***<br>**(−10.22,−4.37)**	**−9.15***<br>**(−12.16,−6.15)**	**−14.13***<br>**(−19.68,−8.58)**
Triglycerides [c]	**−1.56***<br>**(−2.05,−1.08)**	**−2.43***<br>**(−3.00,1.86)**	**−3.46***<br>**(−4.56,−2.36)**	**−2.75***<br>**(−3.64,−1.86)**	**−3.32***<br>**(−4.50,−2.14)**	**−5.81***<br>**(−8.00,−3.61)**	**−6.64***<br>**(−8.93,−4.35)**	**−8.82***<br>**(−11.74,−5.89)**	**−13.00***<br>**(−18.67,−7.29)**
TyG index	**−0.019***	**−0.021***	**−0.025***	**−0.029***	**−0.026***	**−0.035***	**−0.074***	**−0.062***	**−0.091***

	**(−0.024,−0.013)**	**(−0.026,−0.017)**	**(−0.031,−0.020)**	**(−0.038,−0.020)**	**(−0.034,−0.017)**	**(−0.046,−0.024)**	**(−0.100,−0.050)**	**(−0.085,−0.040)**	**(−0.120,−0.062)**
TyG index [c]	**−0.014***	**−0.018***	**−0.023***	−0.023	**−0.025***	**−0.034***	**−0.062***	**−0.067***	**−0.084***
	**(−0.018,−0.009)**	**(−0.023,−0.013)**	**(−0.029,−0.018)**	(−0.030,−0.016)	**(−0.034,−0.016)**	**(−0.044,−0.024)**	**(−0.080,−0.043)**	**(−0.091,−0.044)**	**(−0.110,−0.058)**
TG/HDL ratio	**−0.041***	**−0.068***	**−0.096***	**−0.062***	**−0.094***	**−0.133***	**−0.178***	**−0.254***	**−0.369**
	**(−0.056,−0.025)**	**(−0.086,−0.051)**	**(−0.128,−0.064)**	**(−0.092,−0.033)**	**(−0.127,−0.060)**	**(−0.198,−0.067)**	**(−0.253,−0.100)**	**(−0.338,−0.170)**	**(−0.537,−0.200)**
TG/HDL ratio [c]	**−0.038***	**−0.058***	**−0.077***	**−0.061***	**−0.094***	**−0.122***	**−0.164***	**−0.242***	**−0.316***
	**(−0.050,−0.026)**	**(−0.076,−0.040)**	**(−0.109,−0.045)**	**(−0.085,−0.037)**	**(−0.128,−0.060)**	**(−0.187,−0.058)**	**(−0.226,−0.102)**	**(−0.332,−0.152)**	**(−0.480,−0.152)**
Visceral Adiposity Index	**−0.028***	**−0.054***	**−0.079***	**−0.041***	**−0.075***	**−0.090***	**−0.119***	**−0.195***	**−0.245***
	**(−0.040,−0.016)**	**(−0.069,−0.040)**	**(−0.104,−0.055)**	**(−0.064,−0.018)**	**(−0.100,−0.049)**	**(−0.138,−0.042)**	**(−0.178,−0.059)**	**(−0.261,−0.129)**	**(−0.364,−0.130)**
Visceral Adiposity Index[c]	**−0.030***	**−0.048***	**−0.062***	**−0.039***	**−0.075***	**−0.091***	**−0.111***	**−0.202***	**−0.228***
	**(−0.041,−0.019)**	**(−0.062,−0.034)**	**(−0.086,−0.039)**	**(−0.058,−0.019)**	**(−0.102,−0.049)**	**(−0.138,−0.043)**	**(−0.163,−0.059)**	**(−0.270,−0.134)**	**(−0.352,−0.105)**
Insulin[e]	**−0.003***	**−0.004***	**−0.008***	−0.003	−0.002	−0.007	−0.006	−0.005	−0.018
	**(−0.005,−0.001)**	**(−0.007,−0.001)**	**(−0.014,−0.003)**	(−0.007,0.001)	(−0.008,0.003)	(−0.018,0.003)	(−0.017,0.006)	(−0.020,0.009)	(−0.047,0.011)
Insulin [c,e]	−0.001	−0.002	−0.004	−0.0005	0.001	−0.001	−0.001	0.005	−0.0005
	(−0.003,0.001)	(−0.005,0.001)	(−0.009,0.0002)	(−0.004,−0.003)	(−0.004,0.007)	(−0.010,0.007)	(−0.011,0.008)	(−0.009,0.018)	(−0.023,0.022)
HOMA−IR [e]	**−0.021***	**−0.038***	**−0.092***	−0.021	−0.031	−0.082	−0.046	−0.066	−0.206
	**(−0.037,−0.004)**	**(−0.060,−0.016)**	**(−0.137,−0.047)**	(−0.053,0.010)	(−0.072,0.009)	(−0.165,0.002)	(−0.130,0.038)	(−0.173,0.041)	(−0.428,0.016)
HOMA−IR [c,e]	−0.009	**−0.023***	−0.036	−0.016	−0.005	−0.006	−0.031	0.009	−0.013
	(−0.024,0.006)	**(−0.044,−0.002)**	(−0.074,0.002)	(−0.043,0.012)	(−0.044,0.035)	(−0.078,0.067)	(−0.103,0.041)	(−0.096,0.115)	(−0.205,0.179)

[a] Serum free and bioavailable 25(OH)D haplotype adjusted considering the specific binding coefficients for each one of the six possible phenotypes of VDBP. b Models were adjusted for sex, age groups, the season of serum sampling, vitamin D consumption, leisure−time physical activity, smoking status. c Additional adjustment by BMI. d TyG: triglycerides and glucose index. e Data in a subsample of 1,263 individuals.

Table S5. Association between total, free and bioavailable 25(OH)D levels, adiposity markers and metabolic traits in the Health Workers Cohort Study.

Outcome	Free 25–(OH)D (pg/mL)	*P*–value	Bioavailable 25–(OH)D (ng/mL)	*P*–value
	OR (95% CI)		OR (95% CI)	
BMI				
Normal	Ref.		Ref.	
Overweight	0.95(0.91,0.99)	0.047	0.85(0.75,0.96)	0.008
Obesity	0.93(0.88,0.98)	0.009	0.75(0.64,0.87)	<0.001
Body fat proportion				
Tertil 1	Ref.		Ref.	
Tertil 2	0.96(0.90,1.03)	0.257	0.86(0.73,1.02)	0.088
Tertil 3	0.94(0.87,1.01)	0.082	0.77(0.63,0.92)	0.005
Metabolic syndrome– ATP III				
No	Ref.		Ref.	
Yes	0.89(0.86,0.94)	<0.001	0.73(0.65,0.82)	<0.001
Metabolic syndrome– ATP III [a]				
No	Ref.		Ref.	
Yes	0.90(0.86,0.95)	<0.001	0.77(0.68,0.87)	<0.001
High waist circumference				
No	Ref.		Ref.	
Yes	0.95(0.91,0.99)	0.031	0.82(0.73,0.93)	0.001
Type 2 diabetes				
No	Ref.		Ref.	
Impaired glucose tolerance	0.98(0.94,1.03)	0.431	0.99(0.87,1.11)	0.831
Yes	0.88(0.82,0.94)	<0.001	0.72(0.60,0.86)	<0.001
Type 2 diabetes [a]				
No	Ref.		Ref.	
Impaired glucose tolerance	0.99(0.94,1.04)	0.708	1.03(0.91,1.16)	0.669
Yes	0.88(0.83,0.95)	0.001	0.75(0.63,0.90)	0.002
Elevated blood pressure				
No	Ref.		Ref.	
Yes	0.97(0.92,1.01)	0.136	0.93(0.83,1.05)	0.255
Elevated blood pressure [a]				

No	Ref.		Ref.	
Yes	0.98(0.94,1.03)	0.393	0.99(0.87,1.11)	0.827
Low HDL−C				
No	Ref.		Ref.	
Yes	0.97(0.93,1.01)	0.148	0.87(0.78,0.97)	0.010
Low HDL−C [a]				
No	Ref.		Ref.	
Yes	0.98(0.94,1.02)	0.312	0.90(0.80,0.99)	0.048
High triglycerides				
No	Ref.		Ref.	
Yes	0.83(0.79,0.87)	<0.001	0.63(0.56,0.70)	<0.001
High triglycerides [a]				
No	Ref.		Ref.	
Yes	0.83(0.80,0.87)	<0.001	0.64(0.57,0.72)	<0.001
TyG index [b]				
Tertil 1	Ref.		Ref.	
Tertil 2	0.91(0.86,0.95)	<0.001	0.76(0.67,0.87)	<0.001
Tertil 3	0.80(0.75,0.84)	<0.001	0.59(0.51,0.67)	<0.001
TyG index [a,b]				
Tertil 1	Ref.		Ref.	
Tertil 2	0.91(0.87,0.96)	<0.001	0.78(0.69,0.89)	<0.001
Tertil 3	0.80(0.75,0.85)	<0.001	0.61(0.52,0.70)	<0.001
TG/HDL ratio				
Tertil 1	Ref.		Ref.	
Tertil 2	0.92(0.88,0.97)	0.001	0.78(0.69,0.89)	<0.001
Tertil 3	0.86(0.82,0.91)	<0.001	0.68(0.60,0.78)	<0.001
TG/HDL ratio [a]				
Tertil 1	Ref.		Ref.	
Tertil 2	0.93(0.88,0.97)	0.003	0.80(0.71,0.92)	0.001
Tertil 3	0.87(0.82,0.92)	<0.001	0.71(0.62,0.82)	<0.001
Visceral adipositity Index (VAI)				
Tertil 1	Ref.		Ref.	
Tertil 2	0.92(0.88,0.97)	0.001	0.78(0.68,0.88)	<0.001

Tertil 3	0.81(0.77,0.86)	<0.001	0.58(0.50,0.67)	<0.001
Visceral adiposity Index (VAI) [a]				
Tertil 1	Ref.		Ref.	
Tertil 2	0.92(0.88,0.97)	0.002	0.79(0.70,0.90)	<0.001
Tertil 3	0.82(0.77,0.87)	<0.001	0.60(0.52,0.69)	<0.001
HOMA−IR (>3.2) [c]				
No	Ref.		Ref.	
Yes	0.93(0.87,0.98)	0.008	0.79(0.68,0.92)	0.002
HOMA−IR (>3.2) [ac]				
No	Ref.		Ref.	
Yes	0.93(0.88,0.99)	0.029	0.83(0.71,0.98)	0.029

All models were adjusted for sex, age groups, the season of serum sampling, vitamin D consumption, leisure−time physical activity, smoking status. [a] Additional adjustment by BMI. [b]TyG: triglycerides and glucose index. [c] Data in a subsample of 1,263 individuals.

Table S6. Quantile regression results for the different percentiles of adiposity markers and metabolic traits in the Health Workers Cohort Study.

Outcome	Free 25(OH)D (pg/mL) Coefficient (95% CI)			Bioavailable 25(OH)D SNP (ng/mL) Coefficient (95% CI)		
	25th	50th	75th	25th	50th	75th
BMI	**−0.12* (−0.22,−0.02)**	**−0.17* (−0.27,−0.07)**	−0.14 (−0.29,0.01)	**−0.43* (−0.69, −0.16)**	**−0.59* (−0.86,−0.32)**	**−0.58* (−0.97,−0.19)**
Body fat proportion	**−0.33* (−0.53,−0.14)**	**−0.29* (−0.44,−0.15)**	**−0.22* (−0.39,−0.06)**	**−0.96 (−1.46, −0.45)**	**−0.87* (−0.16, −0.49)**	**−0.74* (−1.18,−0.31)**
Waist circumference	**−0.32* (−0.58,−0.06)**	**−0.49* (−0.74,−0.24)**	−0.26 (−0.57,0.06)	**−1.25* (−1.93, −0.57)**	**−1.42* (−2.04,−0.78)**	**−1.41* (−2.28,−0.54)**
Glucose	**−0.26* (−0.50,−0.03)**	**−0.38* (−0.64,−0.11)**	**−0.66* (−1.19,−0.14)**	−0.56 (−1.15,0.05)	**−0.88* (−1.58,−0.18)**	**−1.70* (−3.1,−0.3)**
Glucose [a]	−0.07 (−0.28.0.14)	−0.31* (−0.56,−0.05)	−0.58* (−1.11,−0.05)	−0.00 (−0.52,0.52)	−0.55 (−1.22,0.13)	−1.27 (−2.68,0.12)
Diastolic blood pressure	−0.10 (−0.35,0.15)	−0.02 (−0.28,0.24)	−0.12 (−0.40,0.17)	−0.38 (−1.03,0.27)	−0.06 (−0.72,0.60)	−0.30 (−1.04,0.44)
Diastolic blood pressure [a]	−0.07 (−0.33,0.18)	−0.01 (−0.26,0.24)	−0.05 (−0.33,0.23)	−0.07 (−0.73,0.59)	0.04 (−0.61,0.69)	−0.11 (−0.85,0.63)
Systolic blood pressure	−0.10 (−0.45,0.24)	−0.34 (−0.70,0.03)	**−0.52* (−0.93,6.09)**	−0.30 (−1.18,0.60)	−0.76 (−1.71,0.18)	**−1.27* (−2.35,−0.19)**
Systolic blood pressure [a]	−0.09 (−0.42,0.23)	−0.24 (−0.59,0.12)	−0.37 (−0.80,0.05)	−0.16 (−1.00,0.68)	−0.35 (−1.27,0.57)	−0.69 (−1.79,0.42)
HDL−c	−0.08 (−0.31,0.15)	−0.00 (−0.25,0.25)	0.23 (−0.08,0.54)	0.03 (−0.60,0.65)	0.25 (−0.40,0.90)	**1.14* (0.34,1.93)**
HDL−c [a]	−0.11 (−0.34,0.12)	−0.07 (−0.35,0.21)	0.29 (−0.03,0.61)	−0.04 (−0.64,0.57)	0.07 (−0.65,0.80)	**0.89* (0.06,1.72)**
Triglycerides	**−5.35* (−6.87, −3.83)**	**−6.80* (−8.49, −5.11)**	**−9.20* (−12.24, −6.16)**	**−13.65 (−17.56, −9.75)**	**−16.41* (−20.55, −12.30)**	**−23.29* (−31.44, −15.13)**
Triglycerides [a]	**−4.99* (−6.30, −3.68)**	**−6.05* (−7.72, −4.39)**	**−8.20* (−11.27, −5.12)**	**−10.72* (−14.18, −7.26)**	**−15.65* (−19.89, −11.42)**	**−21.95* (−30.09, −13.81)**
TyG index [b]	**−0.05* (−0.06, −0.03)**	**−0.05* (−0.06, −0.04)**	**−0.06* (−0.08, −0.05)**	**−0.13* (−0.17, −0.09)**	**−0.13* (−0.16, −0.09)**	**−0.17* (−0.21, −0.12)**

TyG index [a,b]	**−0.04* (−0.05,−0.03)**	**−0.05* (−0.06,−0.03)**	**−0.06* (−0.07,−0.04)**	**−0.10* (−0.13,−0.07)**	**−0.12* (−0.15,−0.08)**	**−0.14* (−0.17,−0.10)**
TG/HDL ratio	**−0.11* (−0.16,−0.07)**	**−0.16* (−0.21,−0.11)**	**−0.21* (−0.30,−0.12)**	**−0.31* (−0.42,−0.20)**	**−0.43* (−0.57,−0.30)**	**−0.62* (−0.84,−0.39)**
TG/HDL ratio [a]	**−0.11* (−0.14,−0.07)**	**−0.16* (−0.20,−0.11)**	**−0.19* (−0.29,−0.10)**	**−0.26* (−0.35,−0.17)**	**−0.40* (−0.52,−0.27)**	**−0.51* (−0.74,−0.27)**
Visceral Adiposity Index	**−0.08* (−0.12,−0.05)**	**−0.12* (−0.16,−0.09)**	**−0.16* (−0.22,−0.09)**	**−0.24* (−0.32,−0.16)**	**−0.34* (−0.44,−0.24)**	**−0.45* (−0.63,−0.28)**
Visceral Adiposity Index [a]	**−0.08* (−0.11,−0.05)**	**−0.13* (−0.17,−0.10)**	**−0.13* (−0.20,−0.07)**	**−0.22* (−0.29,−0.14)**	**−0.32* (−0.43,−0.22)**	**−0.37* (−0.55,−0.19)**
Insulin [c]	**−0.007* (−0.013,−0.001)**	**−0.009* (−0.018,−0.001)**	**−0.002* (−0.036,−0.007)**	**−0.022* (−0.038,−0.005)**	**−0.025* (−0.045,−0.003)**	**−0.059* (−0.100,−0.019)**
Insulin [a,c]	−0.005 (−0.010,0.001)	−0.006 (−0.013,0.002)	−0.012 (−0.023,0.001)	−0.010 (−0.024,0.004)	−0.011 (−0.031,0.008)	−0.023 (−0.056,0.010)
HOMA−IR [c]	**−0.065* (−0.110,−0.019)**	**−0.091* (−0.150,−0.03)**	**−0.219* (−0.338,−0.100)**	**−0.151* (−0.271,−0.031)**	**−0.256* (−0.409,−0.102)**	**−0.612* (−0.934,−0.290)**
HOMA−IR [a,c]	−0.038 (−0.079,0.002)	**−0.080* (−0.137,−0.022)**	**−0.108* (−0.210,−0.005)**	−0.085 (−0.194,0.024)	**−0.200* (−0.354,−0.041)**	−0.241 (−0.512,0.030)

All models were adjusted for sex, age groups, the season of serum sampling, vitamin D consumption, leisure−time physical activity, smoking status. [a] Additional adjustment by BMI. [b] TyG: triglycerides and glucose index. [c] Data in a subsample of 1,263 individuals.

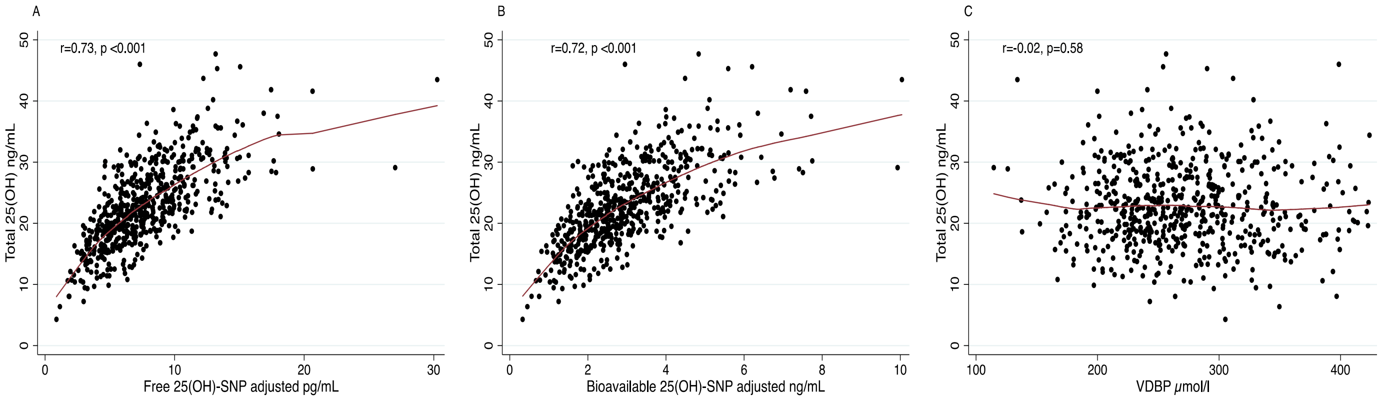

Figure S1. Scatter plot representation of the correlation between total 25(OH)D with free 25(OH)D (A), bioavailable 25(OH)D (B) and VDBP (C) among males from the Health Workers Cohort Study.

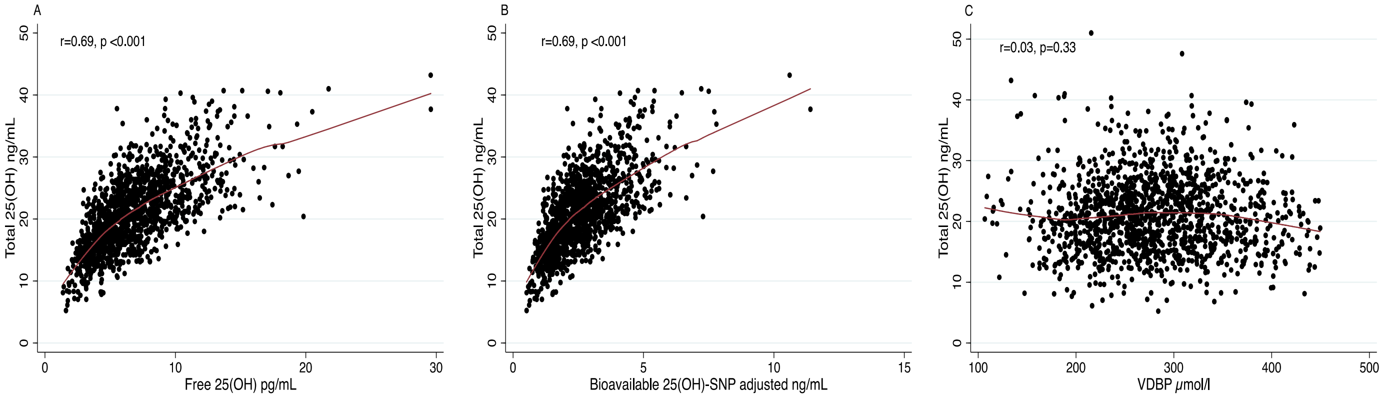

Figure S2. Scatter plot representation of the correlation between total 25(OH)D with free 25(OH)D (A), bioavailable 25(OH)D (B) and VDBP (C) among females from the Health Workers Cohort Study.
